# Effects of balance, coordination, and strength training in children with low vision: a randomized controlled study

**DOI:** 10.1590/1984-0462/2026/44/2025242

**Published:** 2026-08-07

**Authors:** Server Erdoğmuş Gülcan, Erdoğan Kavlak

**Affiliations:** a Mudanya University, Faculty of Health Sciences, Department of Physiotherapy and Rehabilitation, Bursa, Turkey.; b Pamukkale University, Faculty of Physiotherapy and Rehabilitation, Department of Physiotherapy and Rehabilitation, Denizli, Turkey

**Keywords:** Low vision, Pediatric physiotherapy, Balance training, Muscle strength, Motor proficiency, Baixa visão, Fisioterapia pediátrica, Treinamento de equilíbrio, Força muscular, Proficiência motora

## Abstract

**Objective:**

To investigate the effects of an eight-week physiotherapy program focusing on balance, coordination, and muscle strengthening on gait, mobility, posture, and motor proficiency in children with low vision.

**Methods:**

Twenty-two children aged 7-14 years with ophthalmologist-diagnosed low vision, based on World Health Organization criteria, were randomly assigned to treatment (n=10) or control (n=12) groups. The treatment group completed three 75-minute sessions per week for eight weeks, including balance, coordination, and muscle-strengthening exercises. Outcomes were assessed using the BOT-2 Short Form, New York Posture Assessment Test, Lovett muscle grading, and BTS G-Walk. Statistical analyses were performed using Statistical Package for the Social Sciences, version 25.0.

**Results:**

The treatment group showed significant pre- to post-intervention gains in total BOT-2 SF scores and in subscales related to fine motor integration, bilateral coordination, running speed, agility, and strength (p<0.05), with small to moderate effect sizes (Cohen’s d ≈ 0.30-0.90). The control group demonstrated smaller improvements, significant only in some subscales. Postural alignment increased significantly in both groups, with greater effect observed in the treatment group (Cohen’s d=0.74) (p<0.05). Gait analysis revealed increases in left step length and right pelvic propulsion only in the treatment group (p<0.05), whereas no significant changes were observed in the remaining spatiotemporal gait parameters. Manual muscle testing showed strength gains in both groups, with no statistically significant differences between them.

**Conclusions:**

An eight-week, thrice-weekly physiotherapy-based balance, coordination, and strengthening program improves motor proficiency, posture, gait, and muscle strength in children with low vision, supporting its use in pediatric rehabilitation.

## RESUMO

**Objetivo:**

Investigar os efeitos de um programa de fisioterapia de oito semanas com foco em equilíbrio, coordenação e fortalecimento muscular sobre a marcha, mobilidade, postura e proficiência motora em crianças com baixa visão.

**Métodos:**

Vinte e duas crianças de 7 a 14 anos com baixa visão diagnosticada por oftalmologista, de acordo com os critérios da Organização Mundial da Saúde, foram alocadas aleatoriamente nos grupos de tratamento (n=10) e controle (n=12). O grupo de tratamento realizou três sessões semanais de 75 minutos durante oito semanas, incluindo exercícios de equilíbrio, coordenação e fortalecimento muscular. Os desfechos foram avaliados por meio do BOT-2 Short Form, do New York Posture Assessment Test, da graduação muscular de Lovett e do sistema BTS G-Walk. As análises estatísticas foram realizadas no *Statistical Package for the Social Science*, versão 25.0.

**Resultados:**

O grupo de tratamento apresentou ganhos significativos do pré para o pós-intervenção nos escores totais do BOT-2 SF e em subescalas relacionadas à integração motora fina, coordenação bilateral, velocidade de corrida, agilidade e força (p<0,05), com tamanhos de efeito pequenos a moderados (d de Cohen ≈ 0,30-0,90). O grupo controle apresentou melhorias menores, significativas apenas em algumas subescalas. O alinhamento postural apresentou aumento significativo em ambos os grupos, com maior efeito observado no grupo de tratamento (d de Cohen=0.74) (p<0,05). A análise da marcha revelou aumentos no comprimento do passo esquerdo e na propulsão pélvica direita apenas no grupo de tratamento (p<0,05), sem alterações significativas nos demais parâmetros espaço-temporais da marcha. A avaliação muscular manual mostrou ganhos de força em ambos os grupos, sem diferenças estatisticamente significativas entre eles.

**Conclusões:**

Um programa de fisioterapia de oito semanas, realizado três vezes por semana e baseado em exercícios de equilíbrio, coordenação e fortalecimento, melhora a proficiência motora, a postura, a marcha e a força muscular em crianças com baixa visão, apoiando sua aplicação na reabilitação pediátrica.

## INTRODUCTION

Vision is a multidimensional process involving higher-order functions beyond visual acuity, yet visual impairment is primarily assessed using measures such as the Snellen chart.^1^ According to recent World Health Organization (WHO) estimates, at least 2.2 billion people worldwide have near or distance visual impairment, with approximately 1 billion cases being preventable or untreated.^2^ Childhood-specific data are limited; however, regional meta-analyses estimate visual impairment in approximately 19 million children, including 1.4 million who are blind.^3^ Although the global prevalence of blindness and visual impairment declined between 1990 and 2019, the absolute number of affected individuals increased due to population growth.^4^

The etiology of childhood visual impairment varies according to region and socioeconomic status. Uncorrected refractive errors, cataracts, congenital anomalies, corneal scarring, retinopathy, and prematurity-related disorders are the most common causes worldwide. Uncorrected refractive errors predominate in low- and middle-income countries, whereas genetic and congenital factors are more prevalent in high-income settings.^5^

Visual impairment affects not only visual processing but also motor development, balance, muscle strength, posture, and overall movement skills.^6^ Children with visual impairment often show delays in fundamental motor skills such as sitting, standing, balance, and walking, largely due to reduced sensorimotor input and limited opportunities for movement exploration.^7^

Exercise-based and structured motor interventions have been shown to improve balance, posture, coordination, and gait in children with visual impairment, with vestibular-based approaches demonstrating particular benefits.^8^ These effects are attributed to vestibular-proprioceptive integration and neural plasticity, facilitating improved sensorimotor integration and motor control.^9^ ^,^ ^10^

Despite this evidence, most studies focus on isolated outcomes, and comprehensive physiotherapy programs integrating balance, coordination, and muscle strengthening, together with objective gait analysis and standardized motor assessments, remain limited. Therefore, this study aimed to evaluate the effectiveness of a physiotherapy program in gait, mobility, and posture in children with low vision.

## METHOD

**Figure 1.**
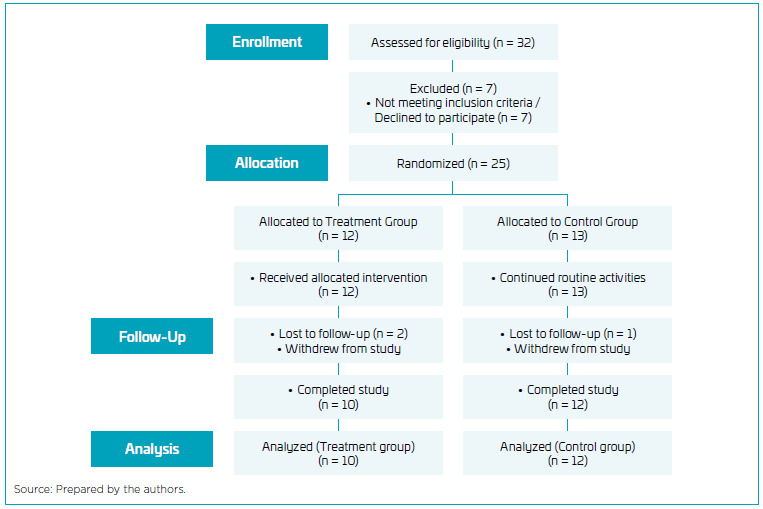
CONSORT flow diagram showing recruitment, allocation, follow-up, and analysis of participants.

This study is a randomized, open, controlled clinical trial conducted in children aged 7-14 years with low vision. The study was designed, conducted, and reported in accordance with the Consolidated Standards of Reporting Trials (CONSORT) Statement. Participant flow through the study is shown in Figure 1. Ethical approval was obtained from the Scientific Research and Publication Ethics Committee of Pamukkale University (dated 26.10.2022, reference no: E-76351742-600-278459). Written informed consent was obtained from parents, and age-appropriate assent was obtained from the participating children.

Initially, 12 children were enrolled in the treatment group and 13 in the control group, representing 78% of the 32 students at the Merkezefendi School for the Visually Impaired. During the study, two participants in the treatment group and one in the control group withdrew, resulting in a final sample of 22 participants (10 treatment, 12 control). An intention-to-treat analysis was planned to minimize bias arising from participant dropouts. The study was conducted between July 2022 and June 2023. Participants were recruited voluntarily from the Denizli/Merkezefendi School for the Visually Impaired (Primary and Secondary) under the Ministry of National Education, Republic of Turkey.

The inclusion criteria were:

1. Ophthalmologist-diagnosed low vision, defined as visual acuity worse than 6/18 in the better eye according to WHO criteria;
2. Absence of neuromuscular or orthopedic conditions preventing physical activity; and
3. Sufficient cognitive ability to follow instructions.

The exclusion criteria were assessed via caregiver reports and medical records, including severe hearing loss, epilepsy, or behavioral disorders that could interfere with participation.

The participants were randomly assigned to two groups using computer-generated simple randomization, stratified by sex and age range to ensure balanced distribution between groups. The intervention consisted of eight weeks of structured physiotherapy, with three 75-minute sessions per week. The eight-week duration was based on established pediatric physiotherapy protocols, and three sessions per week were deemed sufficient to promote meaningful motor adaptation. This schedule aligns with previous research demonstrating the effectiveness of structured, moderate-frequency physiotherapy interventions in enhancing motor skills and postural control in children with visual impairment. The exercises were adapted for developmental levels, but the core program was identical for all participants. All intervention sessions were conducted by the same physiotherapist to ensure consistency. Parents were instructed to maintain their children’s usual daily activities, and no formal recording of extra-intervention motor activities was conducted. The program comprised warm-up, balance, coordination, strengthening, and cool-down components. The control group received no physiotherapy and continued their routine activities. Designed by a physiotherapist based on the literature and adapted to participants’ developmental level, the program included balance board exercises, static and dynamic balance tasks, sandbag strengthening, and proprioceptive stimulation exercises.

The warm-up phase lasted 10 minutes and included 5 minutes of light-paced walking and 5 minutes of stretching targeting the gastrocnemius, hamstrings, hip flexors, and lumbar extensors, with three repetitions per muscle group (15-second stretch, 5-second relaxation). Strengthening exercises involved 0.5 kg sandbags for the first four weeks and 1 kg for weeks 5-8, covering all trunk and limb movement directions, initially with one set of 5 repetitions per exercise and 5-second contraction holds. Repetitions were increased to eight per exercise during weeks 4-8. Coordination training was 20 minutes per session, using Frenkel exercises. In weeks 1-4, exercises were performed with eyes open; in weeks 5-8, exercises were performed with eyes closed. Initially, one set of 5 repetitions was applied, which was increased to eight in the latter half. Balance training also lasted 20 minutes and was performed on a 30×20×300 cm balance bar and a 10 m walking path, with progression occurring only after task completion. The cool-down mirrored the warm-up stretches to ensure a structured rehabilitation session. Program adherence was monitored via attendance records, with session completion documented. Parents were instructed to maintain their usual physical activity and screen-time routines, and sociodemographic and health data were collected using a standardized form. Motor proficiency was assessed using the Bruininks-Oseretsky Test of Motor Proficiency Short Form (BOT-2 SF), which evaluates fine motor skills, coordination, balance, running speed, and strength within 15-20 minutes. The short form was selected to minimize assessment time and participant fatigue while retaining acceptable reliability and validity for children with low vision.^11^ The BOT-2 SF total scores range from 0-100, with higher scores indicating better overall motor proficiency, encompassing fine and gross motor skills, balance, coordination, and strength. Posture was assessed using the New York Posture Assessment Test, which scores 13 body regions on a 1-5 scale (total score: 13-65).^12^ Muscle strength was evaluated with the Lovett method (0-5) based on movement against gravity and resistance.^13^ Gait analysis was performed using the BTS G-Walk System, providing spatiotemporal, spinal, and pelvic kinematic parameters with automated comparison to normative data.^14^ All assessments followed standardized protocols, and intra- and inter-rater reliability were established prior to data collection.

The sample size calculation was based on the total scores of the New York Posture Assessment Test. In the reference study, a large effect size (Cohen’s d=2.38) was reported for this measure.^15^ For the sample size calculation, a more conservative approach was adopted, using an effect size of d=1.4. Based on this calculation, 20 participants (at least 10 per group) were estimated to provide 80% power at a 95% confidence level. Data were analyzed using the Statistical Package for the Social Sciences (SPSS), version 25.0. Continuous variables are presented as mean±standard deviation, categorical variables as number and percentage. Effect sizes (Cohen’s d) and 95% confidence intervals were calculated to complement p-values and enhance clinical interpretability. The normality of continuous data was assessed using the Shapiro-Wilk test. Parametric assumptions determined independent t-tests or Mann-Whitney U tests for intergroup comparisons, paired t-tests or Wilcoxon signed-rank tests for dependent groups, and ꭓ^2^ tests for categorical variables, with p<0.05 considered significant.^16^ Comparisons between the treatment and control groups were performed without further adjustment, as the groups were matched for sex, age, height, body mass index, and degree of visual impairment at baseline.

## RESULTS

The mean age was 11.5±1.9 years in the treatment group and 10.17±2.66 years in the control group. Mean body mass index was 17.92±3.39 kg/m^2^ and 17.75±2.61 kg/m² in the treatment and control groups, respectively. Additional participant characteristics for both groups are presented in Table 1.

**Table 1.** Comparison of age, height, weight, and body mass index variables by groups.

	n	Mean±SD	MD (95%CI)	p-value
Age				
Treatment	10	11.50±1.90	1.33 (-1.02; 2.55)	0.314
Control	12	10.17±2.66		
Length				
Treatment	10	148.80±16.59	6.80 (-5.30; 18.90)	0.418
Control	12	142.00±17.38		
Weight				
Treatment	10	41.00±14.76	4.25 (-8.12; 16.62)	0.469
Control	12	36.75±12.29		
BMI				
Treatment	10	17.92±3.39	0.17 (-2.01; 2.35)	0.896
Control	12	17.75±2.61		

n: number of cases; SD: standard deviation; MD: mean difference (treatment, control); 95%CI: 95% confidence interval of the mean difference; BMI: body mass index. Independent samples t-test was used.

Source: Prepared by the authors.

Comparison of the BOT-2 SF scores before and after the intervention showed a statistically significant increase in total scores in both the treatment and control groups (p<0.05). Examination of the BOT-2 SF subscales revealed that in the control group, fine motor integration, bilateral coordination, and strength subscale scores increased significantly. In the treatment group, significant improvements were observed in fine motor integration, bilateral coordination, running speed and agility, and strength subscales, as well as in composite scores representing body coordination, running speed and agility, and strength and agility (p<0.05) (Table 2).

**Table 2.** Comparison of Bruininks-Oseretsky Motor Competence subtest scores of the treatment and control groups before and after treatment.

Test	Group	Before Treatment (Mean±SD)	After Treatment (Mean±SD)	MD (95%CI)	p-value	Cohen’s d
Fine motor precision	Treatment	6.83±5.89	6.58±6.24	-0.25 (-1.81; 1.31)	0.703	0.11
	Control	6.30±2.67	6.00±2.11	-0.30 (-1.42; 0.82)	0.596	
Fine motor integration	Treatment	5.25±4.09	6.25±4.65	1.00 (-0.20; 2.20)	0.041*	-0.09
	Control	5.90±2.38	7.00±2.40	1.10 (-0.05; 2.25)	0.026*	
Manual dexterity	Treatment	4.08±2.97	4.25±2.67	0.17 (-0.69; 1.03)	1	0.03
	Control	4.10±1.52	4.10±1.52	0.00 (-0.47; 0.47)	0.480	
Bilateral coordination	Treatment	4.33±3.08	6.17±2.12	1.84 (-3.40; -0.25)	0.041*	0.09
	Control	4.80±2.44	6.70±1.49	1.90 (-3.63; -0.16)	0.041*	
Balance	Treatment	5.42±2.87	6.08±2.35	0.66 (-1.72; 0.39)	0.298	-0.02
	Control	4.60±1.78	5.30±1.25	0.70 (-2.13; 0.73)	0.194	
Running speed and agility	Treatment	5.25±3.39	5.67±3.68	0.42 (-1.78; 0.95)	0.041*	-0.21
	Control	4.80±2.86	5.90±2.85	1.10 (-2.13; -0.06)	0.516	
Upper limb coordination	Treatment	4.92±5.52	4.75±4.99	-0.17 (-0.48; 0.82)	0.124	-0.51
	Control	2.30±2.87	4.10±2.64	1.80 (-4.20; 0.60)	0.516	
Strength	Treatment	5.58±3.06	7.08±1.78	1.50 (-2.89; -0.10)	0.048*	0.00
	Control	4.70±2.63	6.20±1.75	1.50 (-2.97; -0.02)	0.037*	
Total Score	Treatment	41.67±28.39	46.83±24.35	5.16 (-10.39; 0.05)	0.013*	-0.14
	Control	37.70±12.16	45.30±8.14	7.60 (-13.18; -2.01)	0.050*	
Fine manual control	Treatment	12.08±9.83	12.83±10.03	0.75 (-2.66; 1.16)	0.393	-0.01
	Control	12.20±4.34	13.00±4.14	0.80 (-2.81; 1.21)	0.408	
Manual coordination	Treatment	8.83±7.99	9.00±7.31	0.17 (-1.01; 0.68)	0.115	-0.34
	Control	6.30±3.47	8.20±2.15	1.90 (-4.36; 0.56)	0.674	
Body coordination	Treatment	9.75±5.72	12.25±4.35	2.50 (-4.96; -0.03)	0.032*	-0.32
	Control	9.40±3.57	12.00±2.45	2.60 (-4.91; -0.28)	0.058	
Strength and agility	Treatment	10.83±5.92	12.75±4.99	1.92 (-3.95; 0.11)	0.026*	-0.04
	Control	9.50±4.88	12.10±4.18	2.60 (-4.81; -0.38)	0.063	

*p<0.05 statistically significant difference.

SD: standard deviation; MD: mean difference (treatment, control); 95%CI: 95% confidence interval of the mean difference; Cohen’s d values represent the effect size of the difference between the treatment and control groups from pre- to post-intervention for each subtest.

Source: Prepared by the authors.

Spatiotemporal gait parameters were assessed using the BTS G-Walk Wireless Digital Gait Analysis System. No statistically significant differences were found in cadence, gait speed, right quality, stance phase (SP)-Right, oscillation phase (OP)-Right, single support phase (SSP)-Left, double support phase (DSP)-Right, DSP-Left, and right step length within or between groups, before and after the intervention. Statistically significant parameters are presented in Table 3.

**Table 3.** Comparison of gait characteristics of individuals.

Variable	Treatment Group BT/AT (Mean±SD)	Control Group BT/AT (Mean±SD)	Treatment Group Δ (Mean±SD)	Control Group Δ (Mean±SD)	Treatment Group Recovery (%) (Mean±SD)	Control Group Recovery (%) (Mean±SD)	MD (95%CI)	p-value^1^ BT/AT Δ/ Recovery (%)	p-value^2^ TG/CG	Cohen’s d
Time	27.31±12.13/14.77±4.51	30.82±22.75/19.49±16.42	12.54±10.94	11.33±23.82	-38.92±27.66	-26.99±46.60	1.21 (-13.88; 16.30)	0.821/0.872	0.117/0.006*	0.06
								0.582/0.674		
Left Quality	94.89±3.71/98.02±1.46	95.85±4.6/95.9±3. 23	-3.13±4.26	-0.05±6.01	3.46±4.70	0.31±6.79	-3.08 (-7.77 ; 1.61)	0.539/0.059	0.978/0.045*	-0.58
								0.189/0.107		
SP-Left	61.22±3.02/60.42±1.19	55.94±7.64/59.6±2.65	0.80±2.71	-3.66±9.14	-1.13±4.24	9.88±27.40	4.46 (-1.45 ; 10.37)	0.014*/0.059	0.239/0.878	0.64
								0.418/0.418		
OP-Left	38.79±3.02/39.58±1.19	41.64±2.65/40.4±2.65	-0.80±2.71	1.24±2.72	2.53±7.54	-2.83±6.15	-2.04 (-4.41 ; 0.33)	0.043*/0.059	0.143/0.878	-0.75
								0.418/0.456		
SSP-Right	38.71±3.02/39.8±1.52	41.73±2.61/40.44±2.85	-1.09±2.99	1.29±2.78	3.32±8.36	-2.96±6.36	-2.38 (-8.60 ; 3.84)	0.02*/0.14	0.182/0.279	-0.83
								0.18/0.059		
DSL-Right	1.11±0.27/1.1±0.14	1.02±0.22/1.11±0.23	0.00±0.21	-0.09±0.13	3.48±21.38	9.78±13.77	0.09(-15.16 ; 15.34)	0.424/1	0.032*/0.953	0.53
								0.205/0.413		
DSL-Left	1.1±0.27/1.1±0.13	1.02±0.22/1.12±0.23	0.00±0.22	-0.09±0.13	4.19±22.43	9.91±13.75	0.09(-15.94 ; 16.12)	0.446/0.876	0.029*/1	0.51
								0.226/0.471		
Left Step Length	49.25±1.46/54.5±8.88	50.89±1.84/50.05±1.96	-5.26±9.04	0.84±2.77	10.78±18.42	-1.53±5.29	-6.10 (-17.86 ; 5.66)	0.059/0.283	0.695/0.011*	-0.95
								0.036*/0.036*		
Number of Right Steps	10.8±5.63/8.1±1.1	11.75±5.93/8.25±2.34	2.70±4.81	3.50±4.34	-15.04±23.24	-22.46±20.29	-0.80 (-3.27; 4.87)	0.674/0.674	0.005*/0.058	-0.18
								0.346/0.433		
Number of Left Steps	11±5.91/8.4±1.26	11.58±6.13/8.42±2.57	2.60±5.13	3.17±4.28	-13.24±23.08	19.11±21.98	-0.57 (-3.61; 4.75)	0.872/0.582	0.01*/0.072	-0.12
								0.418/0.549		

*p<0.05 statistically significant difference.

SD: standard deviation; MD: mean difference (treatment, control); 95%CI: 95% confidence interval of the mean difference; p^1^: p-value for difference between independent groups; p^2^: p-value for difference between dependent groups; Δ: Change value between groups before and after treatment; AT: after treatment. BT: before treatment; TG: treatment group; CG: control group; SP: stance phase; OP: oscillation phase; SSP: single support phase; DSL: double stride length. Cohen’s d values represent the effect size of the difference between the treatment and control groups from pre- to post-intervention for each subtest.

Post-intervention manual muscle testing revealed no significant differences between the treatment and control groups in trunk, lower-extremity, or upper-extremity muscle strength. Comparison of pre- and post-intervention scores within each group showed that in the control group, anterior trunk flexors, right and left hip extensors, right and left hip adductors, and right and left shoulder flexors demonstrated significant improvements (p<0.05). In the treatment group, significant increases were observed in back extensors, right and left hip flexors, right and left hip extensors, right hip abductors, right hip adductors, right and left knee extensors, right and left ankle plantar flexors, right and left shoulder flexors, right and left shoulder extensors, right forearm supinators, right forearm pronators, and left forearm pronators (p<0.05).

Regarding postural characteristics assessed with the New York Posture Assessment Test, no significant differences were found between groups before the intervention. However, within-group comparisons before and after the intervention indicated significant improvements in both groups (p<0.05). Post-intervention between-group comparisons revealed a statistically significant difference (p<0.05), while comparisons of pre- to post-intervention changes between groups showed no significant difference. The treatment group also demonstrated a higher percentage of improvement. The New York Posture Assessment Test scores are presented in Table 4.

**Table 4.** Comparison of New York Posture Evaluation Test scores.

Group	BT (Mean±SD)	AT (Mean±SD)	Δ (Mean±SD)	Recovery (%) (Mean±SD)	MD (95%CI)	p-value^1^	p-value^2^	Cohen’s d
Treatment	34.50±4.84	40.80±6.49	-6.30±8.41	36.86±27.96	6.30 (-1.21; 13.27)	0.418	0.040*	0.74
Control	36.33±6.23	48.67±7.08	-12.33±7.85	20.34±25.89	12.33 (-7.85; -1.21)	0.418	0.000*	0.98

New York Posture Assessment Test scoring: ≥45 “very good”; 40-44 “good”; 30-39 “ moderate”; 20-29 “ poor”; ≤19 “ bad”.

*p<0.05 statistically significant difference.

BT: before treatment; AT: after treatment; SD: standard deviation; Δ: Change value between groups before and after treatment. MD: mean difference (treatment, control); 95%CI: 95% confidence interval of the mean difference; p^1^: p-value for difference between independent groups; p^2^: p-value for difference between dependent groups; Cohen’s d values represent the effect size of the difference between the treatment and control groups from pre- to post-intervention for each subtest.

Source: Prepared by the authors.

## DISCUSSION

Children with visual impairments face multiple challenges in motor skill development, with daily movements being slower and requiring more repetitions than in sighted peers. Posture, flexibility, trunk and extremity muscle strength, balance, and gait are variably affected, and physical fitness and exercise tolerance are generally reduced, which may limit participation in physical activity.^17^

In this study, an eight-week balance, coordination, and strengthening training program was implemented three times per week to investigate its effects on gait, mobility, and posture in children with low vision. The program was associated with improvements primarily in postural parameters, while changes in gait-related measures were limited and should be interpreted cautiously. Although no statistically significant between-group differences were observed, the treatment group showed greater within-group improvements in several motor and postural parameters. These parameters may have influenced the magnitude of change, especially in strength and balance components. Future studies employing higher intensity, longer duration, and more frequent sessions may therefore reveal clearer between-group distinctions.

In typically developing individuals, balance relies on the integration and processing of sensory inputs, enabling adaptive motor responses, with coordinated movement being closely associated with balance capacity.^18^ Maintaining balance during walking is particularly important for individuals with visual impairments, as balance deficits may affect walking stability. Beyond behavioral outcomes, motor and postural adaptations in the treatment group are likely driven by neurophysiological mechanisms, including enhanced sensorimotor integration, improved proprioceptive feedback, and task-specific neural reorganization, underscoring the role of sensory-motor interaction in compensating for reduced visual input.^19^

Using the BOT-2, Atasavun et al.^20^ and Uysal and Düger^21^ reported lower balance scores in children with visual impairment compared with typically developing peers, while Atasavun et al.^20^ and Uysal and Düger^21^ observed slower straight-line walking performance in visually impaired children.^20^ ^,^ ^21^ Similarly, in a study of 127 children with low vision or total visual impairment, BOT-2 balance subscale assessments indicated significantly poorer balance in children with total visual impairment than in typically developing peers.^22^ In a study of 103 visually impaired males, goalball athletes demonstrated superior balance performance compared with non-athletes.^23^ A thesis study with 43 visually impaired children aged 10-12 years found significant improvements in balance performance in the treatment group after a 14-week physical activity program compared to control and comparison groups.^24^

These findings indicate that children with visual impairments have lower balance abilities than their typically developing peers, which negatively affect motor performance. Regular physical activities, such as goalball, positively influence balance, underscoring the importance of structured programs for motor skill development. In the present study, BOT-2 SF assessments showed increased body coordination scores in the treatment group, reflecting improvements in bilateral coordination and balance. These results are consistent with the literature, suggesting that balance- and coordination-based interventions can enhance postural control and motor proficiency in this population.

Post-intervention motor and postural adaptations may result from enhanced sensorimotor integration, improved proprioceptive feedback, and task-specific neural reorganization. These neural changes underscore the role of multisensory processing in compensating for visual deficits and enhancing balance performance.

Both groups showed increases in total BOT-2 scores, with the treatment group demonstrating significant gains in fine motor integration, bilateral coordination, running speed, agility, strength, body coordination, and upper extremity coordination. Improvements in the control group likely reflect natural development or repeated test exposure. These findings align with previous research indicating that maturation alone can lead to partial motor gains without structured intervention.

A gait analysis study of 60 children found that those with visual impairment exhibited greater balance and gait difficulties, characterized by shorter step length, wider foot angles, and slower walking speed than their typically developing peers.^21^ Similarly, children and adolescents aged 8-18 years with visual impairment exhibit greater postural sway, shorter single-leg stance times, and lower physical activity levels than normally sighted peers.^25^ These studies highlight significant deficits in gait and balance in visually impaired children and adolescents. Characteristics such as short step length, wide foot angle, and low gait speed reflect difficulties in motor control. Furthermore, poor postural stability and low physical activity negatively impact independent mobility. The balance, coordination, and strengthening program implemented in this study may have contributed to limited improvements in certain gait-related parameters; however, no clear between-group differences were observed, and these findings should be interpreted with caution.

The structured, progressively challenging nature of the program suggests its potential applicability in both school-based physical education and clinical rehabilitation settings. Integrating such programs into educational routines could increase participation in physical activity, foster independence, and improve social inclusion among children with visual impairment.

In a Turkish school-based study of visually impaired students, those participating in sports showed higher mean strength across abdominal, back, hip, leg, and arm muscles than non-participants, with sex-stratified analyses confirming superior strength in all parameters among the sports group.^26^ In a thesis study of 80 hearing-impaired children aged 10-15 years, a 16-week educational sports games program resulted in significant strength gains in the treatment group compared with controls.^27^

These findings suggest that regular physical activity and structured exercise programs enhance muscle strength, with the greater and more widespread post-intervention gains in the treatment group supporting the effectiveness of the strengthening protocol. The program appears feasible in both school-based and clinical rehabilitation settings, whereas the limited improvements in the control group may reflect age-related developmental differences, underscoring the value of early physical activity-based interventions for children with visual impairment.

Studies have also reported prevalent postural deviations among visually impaired children, including anterior or lateral head and neck tilt, thoracic hyperextension, and flexion in the hip and knee joints.^28^ ^,^ ^29^ These compensatory postural patterns reflect inadequate postural control, with features such as absent arm swing, knee-flexed gait, and irregular movements indicating reduced postural stability. Consistent with the literature, the present findings confirm postural alignment challenges during walking in visually impaired children. Greater postural improvements in the treatment group suggest that balance- and coordination-based training facilitates postural adaptation, supporting the integration of sensorimotor training into school routines and clinical rehabilitation to enhance stability and prevent maladaptive movement patterns.

Children with visual impairments are generally less physically active than their sighted peers.^30^ Independent mobility training and the use of assistive devices are critical, with auditory and kinesthetic perception playing a key role. These findings underscore that enhancing independent movement in visually impaired children requires not only increasing physical capacity but also developing perceptual strategies based on auditory and kinesthetic cues. Although participation in physical activity and confidence were not directly assessed in the present study, improvements in balance, coordination, and postural control may support functional mobility, as suggested in previous literature.

This program integrated structured balance, coordination, and strengthening interventions for children with low vision, using objective measures to assess motor proficiency, posture, strength, gait, and mobility. The methodology ensured developmental appropriateness, ethical compliance, and statistical rigor; however, assessments were conducted by a non-blinded physiotherapist, which is acknowledged as a methodological limitation. These results are consistent with the existing literature and support the effectiveness of structured physical activity programs for children with low vision.

Overall, the eight-week, thrice-weekly balance, coordination, and strengthening program positively influenced gait, mobility, and posture in children with low vision. Improvements in posture, bilateral coordination, and balance suggest that multifaceted programs can effectively support motor performance. Non-significant differences in some variables may reflect the limited sample size and short intervention period, highlighting the need for larger, longer-term studies. Limitations such as a small sample size, short duration, and lack of blinding restrict generalizability; thus, the findings should be interpreted cautiously. Future research should evaluate the functional impact of similar programs on daily activities across different age groups and levels of visual impairment to optimize intervention protocols. Such approaches can provide robust evidence to enhance motor skills, independent mobility, and quality of life in children with visual impairment.

## Data Availability

The database that originated the article is available with the corresponding author.
